# Characterizing the Phan Rang Sheep: A First Look at the Y Chromosome, Mitochondrial DNA, and Morphometrics

**DOI:** 10.3390/ani14142020

**Published:** 2024-07-09

**Authors:** Nguyen Ngoc Luong, Huynh Thi Thu Ha, Nguyen Xuan Huy, Bui Van Loi, Nguyen Huu Van, Hoang Tan Quang, Nguyen Hoang Loc

**Affiliations:** 1 College of Sciences, Hue University, 77 Nguyen Hue, Hue 530000, Vietnam; 2 Department of Science, Technology and International Relations, Hue University, 4 Le Loi, Hue 530000, Vietnam; nguyenxuanhuy@hueuni.edu.vn; 3 Faculty of Biology, University of Education, Hue University, 34 Le Loi, Hue 530000, Vietnam; 4 Presidential Board, Hue University, 3 Le Loi, Hue 530000, Vietnam; bvanloi@hueuni.edu.vn; 5 University of Agriculture and Forestry, Hue University, 102 Phung Hung, Hue 530000, Vietnam; nguyenhuuvan@huaf.edu.vn; 6 Institute of Biotechnology, Hue University, Tinh Lo 10, Phu Thuong, Phu Vang 536801, Vietnam; htquang@hueuni.edu.vn

**Keywords:** Vietnamese sheep, Phan Rang sheep breed, D-loop, SRY, SRYM18, maternal lineages, paternal lineages

## Abstract

**Simple Summary:**

Phan Rang sheep is a term used to collectively refer to the sheep raised in the central Vietnam provinces of Ninh Thuan and Binh Thuan. Given the extreme weather conditions of their habitat, Phan Rang sheep are assumed to be heat-resilient. With climate change’s mounting challenges, the sheep have become a focus of government-led breeding and conservation initiatives. However, there is a critical knowledge gap regarding the sheep’s genetic information, including their origins, diversity, population structure, and genetics, as well as the morphological basis of their adaptive traits. This gap has hindered the development of breeding programs and genetic resource conservation efforts. In an effort to contribute to filling this knowledge gap, we employed a two-fold approach to investigate Phan Rang sheep’s morphometrics and genetic lineages. First, we collected body conformational data to facilitate the quick identification of purebred individuals. Secondly, we collected and analyzed the mitochondrial DNA control region (D-loop) and the Y chromosome SRY and SRYM18 sequences to elucidate the maternal and paternal lineages of the sheep. Of the sheep analyzed for the D-loop, approximately one-third belonged to haplogroup A, while the remainder belonged to haplogroup B. For the paternal lineage, two main Y haplotypes, H5 and H6, were identified. Overall, the findings supported the hypothesis of dual origins for the Phan Rang sheep.

**Abstract:**

The Phan Rang sheep, considered the sole indigenous breed of Vietnam, are primarily concentrated in the two central provinces of Ninh Thuan and Binh Thuan, with Ninh Thuan accounting for more than 90% of the country’s sheep population. These provinces are known for their high temperatures and frequent droughts. The long-standing presence of the Phan Rang sheep in these regions suggests their potential resilience to heat stress—a trait of increasing interest in the face of global climate change. Despite the breed’s significance, a critical knowledge gap hinders conservation and breeding programs. To address this, our study employed a two-pronged approach. First, we collected body conformational data to aid in breed identification. Second, we analyzed mitochondrial DNA (D-loop) and Y chromosome markers (SRY and SRYM18) to elucidate the maternal and paternal lineages. Among the 68 Phan Rang sheep analyzed for their D-loop, 19 belonged to mitochondrial haplogroup A, while 49 belonged to haplogroup B. The haplogroups can be subdivided into 16 unique haplotypes. All 19 rams surveyed for their paternal lineages belonged to haplotypes H5 and H6. These findings strongly support the hypothesis of dual origins for the Phan Rang sheep. This study presents the first genetic data for the Phan Rang breed, providing crucial insights for future research and conservation efforts.

## 1. Introduction

Sheep were among the first animals to be domesticated, around 11,000 to 9500 years ago [1]. Following their domestication, sheep have spread globally and demonstrated remarkable adaptability to diverse climatic conditions. This adaptability has resulted in a vast number of breeds, many of which are indigenous to specific regions or nations [2,3]. The sheep’s innate capacity to thrive in extreme environments positions them as a priority for genetic conservation efforts, particularly in light of the escalating challenges posed by climate change [4,5].

The most recent data on Vietnamese livestock indicate that small ruminants, predominantly goats and sheep, number around 3 million, compared to 8.5 million cattle. Of these, approximately 110,000 are sheep [6]. They are largely concentrated in two provinces—Ninh Thuan and Binh Thuan—with Ninh Thuan being the supplier for the breeding stock, hence the name Ninh Thuan sheep, more commonly known among locals as Phan Rang (the name of the capital city) sheep [7]. Ninh Thuan and Binh Thuan are among the two warmest provinces in Vietnam and frequently experience droughts [8,9], which underscore the Phan Rang sheep’s reputation for hardiness and their suitability for genetic conservation efforts. Indeed, data from previous work showed that the growth rates of the Phan Rang sheep raised in different climates in Vietnam, including Ninh Thuan, were roughly similar, and temperatures did not have significant effects on their reproduction characteristics [10,11]. This indirect evidence hints at a heat stress resilience trait. With the growing threat of climate change, policymakers and agriculture scientists are increasingly prioritizing resilient livestock breeds like the Phan Rang sheep for sustainable agricultural strategies.

Historically, Vietnam lacked native sheep, with introductions occurring in the late 19th century. Theories about the Phan Rang sheep’s origins suggest Indian and French influence [7,12,13], but, without genetic evidence, these remain conjectural. Nevertheless, these sheep have thrived in Ninh Thuan’s semi-arid climate for more than 100 years and expanded into Binh Thuan. Efforts to extend their range beyond these two provinces have faced obstacles [10,11]. For communities facing economic hardship, including Cham ethnic minorities, Phan Rang sheep represent a valuable financial reserve, a source of readily available, nutritious meat, providing valuable manure that can be sold for use in ornamental plant cultivation. The situation is similar to many poor communities who also keep sheep in Bangladesh [14].

Facing a serious decline in sheep numbers during the 1990s, the Vietnamese government-initiated imports from West Asia and China to enrich the genetic pool of local sheep [7,13]. It was reported that the efforts met limited success, as most imported sheep failed to acclimate to the region’s extreme conditions [7]. Success was found only recently when the Australian Dorper breed was crossed with the Phan Rang sheep at the Son Tay Goat and Rabbit Research Center. This crossbreeding produced F1 hybrids that not only flourished within the research facility but also adapted well to areas in Ninh Thuan and Binh Thuan [15]. Yet, while these hybrid sheep have flourished, their spread poses a potential threat to the genetic purity and survival of the local Phan Rang sheep, highlighting the need for immediate intervention.

Mitochondrial DNA (mtDNA) markers have gained prominence in the genetic analysis of animal populations, especially for investigating matrilineal inheritance since the pioneering contributions of Avise and Moritz [16,17]. Despite some debate over the ideal characteristics of genetic markers, mtDNA markers are still favored for their simplicity and cost-effectiveness relative to full mitogenome-based population analysis [18]. Commonly employed mtDNA markers include the 12S rRNA, 16S rRNA, D-loop, tRNA, ATPase6/ATPase8, CytB, and COX1 [19,20,21,22]. In sheep population genetic studies, the D-loop is frequently used to trace maternal lineages due to its ideal characteristics for a marker, such as neutrality and high diversity [19,22,23,24,25,26,27]. For the paternal lineages of sheep, researchers often rely on the sex-determining region Y (SRY) and microsatellite M18 of the SRY (SRYM18) markers as representatives for Y chromosome diversity, given the complexity of sequencing the entire Y chromosome in sheep [19,28,29,30,31,32].

Despite the Phan Rang sheep’s cultural and economic importance to the local region, as well as their potential heat-resilient trait, which is extremely valuable for livestock breeding programs in dealing with the challenges posed by climate changes, comprehensive information on the breed is surprisingly elusive. Furthermore, even fundamental information such as breed records and morphometrics is often difficult to obtain, and what can be found tends to be more qualitative than quantitative. Prior work on the Phan Rang sheep has primarily addressed their diet and behavior [33,34] and their adaptation to provinces other than Ninh Thuan and Binh Thuan [10,11]. While the introduction of new sheep breeds has alleviated inbreeding concerns, the rise of hybrids is threatening the genetic integrity of the purebred Phan Rang population. This scarcity of information, coupled with the increasing prevalence of hybrids, underscores the urgent need for a thorough genetic characterization of the Phan Rang sheep.

Our work is unique compared to similar sheep genetic work from other countries. Firstly, this study faced the complete absence of breed records and any official documentation of sheep imports, except for sporadic and vague mentions in some documents. Secondly, the Phan Rang sheep breed concept is poorly defined due to the lack of genetic background information. To shed light on “the breed”, we need to first devise a strategy to investigate the origins of the sheep and then proceed to study the genetic diversity and resilient traits. We also need to take extra precautions when selecting purebred Phan Rang sheep in the context of the rising popularity of hybrids. 

Our study aims to contribute to filling the knowledge gap by providing the first in-depth analysis of the mitochondrial DNA (D-loop) and Y chromosome markers (SRY and SRYM18) in the Phan Rang sheep, along with the morphometric data. The results aim to serve as a starting point for more comprehensive genetic and morphometric studies on the breed to address important questions such as population structure and diversity, environmental and disease resilience mechanisms, and the extent of inbreeding. Additionally, we anticipate that, once the results are made publicly available, they will significantly facilitate later work on the breed by improving the accessibility, visibility, and availability of the Phan Rang sheep information.

## 2. Materials and Methods

### 2.1. Collection of Information on Morphometric Features

We conducted a preliminary assessment of the morphometric characteristics of the Phan Rang sheep. Thirty male and thirty female sheep over 12 months old were sampled. The morphological evaluation included the measurement of various physical parameters: body weight, sternum height, wither height, body length, girth circumference, ear length, cannon bone circumference, and tail length. In addition to these morphometric measurements, we also collected data on fleece textures (fine or coarse) and colors. The data collection was performed with the assistance of certified veterinarians and the research center staff and followed the ethical guidelines for animal research. The data were compared with corresponding data collected previously [11,12,13]. 

### 2.2. Animal Selection and Total DNA Extraction

Blood samples were collected from the Phan Rang sheep by licensed veterinarians in the Ninh Thuan province, specifically from the Ninh Phuoc, Ninh Hai, Thuan Nam, and Bac Ai districts. The sex distribution within the samples mirrored the natural composition of herds in the region (one male–seven females, likely even much lower for breeding sheep), adhering to the local practice of retaining ewes for reproduction and selling rams for meat [35]. Due to the absence of genetic records, we focused on collecting samples from households with a long history of sheep rearing and with herds exceeding 200 sheep. The selection for sampling within each household ranged from five to ten sheep, with the age of the sheep being a key consideration to ensure a representative sample of breeding sires and dams. The selection favored mature breeding adults whenever possible, based on the owners’ records. In addition, to ensure the authenticity of the Phan Rang breed and to rule out potential Dorper crossbreeding, blood was also collected from four Phan Rang and four Dorper dams at the Son Tay Goat and Rabbit Research Center. These samples were subsequently analyzed to determine the breeds’ phylogenetic relationship. The total DNA was isolated using the FavoPrep Blood/Cultured Cell Genomic DNA Extraction Mini Kit (Favorgen, Catalog No FABGK 001-2, Ping Tung, Taiwan). The DNA yields were quantified through agarose gel electrophoresis and complemented by spectrophotometric analysis. 

### 2.3. PCR Amplification and Sequencing

To guarantee the authenticity of the sequence variations and reduce the possibility of artifacts, including sequencing and Taq polymerase errors, we used the Phusion High-Fidelity DNA Polymerase (Thermo Fisher Scientific, Cat No F530S, Waltham, MA, USA) for all the PCR amplification. For the D-loop region, a carefully calibrated quantity of total DNA (25–30 ng) was used to mitigate the risk of the contamination of nuclear mitochondrial DNA segments (NUMTs). To amplify the SRY and SRYM18 markers, we employed 50 to 100 ng of template DNA. The primer sequences are provided in Supplementary Table S2. For the D-loop amplification, the following PCR settings were used: denaturing at 98 °C for 30 s, followed by 30 cycles of denaturing at 98 °C for 10 s, annealing at 58–60 °C for 30 s, and extension at 72 °C for 45 s; the reactions were maintained at 72 °C for 5 min followed by 10 min at 4 °C. The same settings were used for the SRY and SRYM18, except for the extension time being 15 s. Distilled water was used as the template for the negative controls. The PCR products were then purified using the Favorgen Gel/PCR Purification kit (Cat No. FAGCK 00-1, Ping Tung, Taiwan), A-tailed, and cloned into the pGEMT Easy Vector system (Promega, Cat no A1360, Madison, WI, USA) for sequencing. A bidirectional Sanger sequencing was conducted through 1st BASE services (https://base-asia.com/standard-sequencing/, accessed on 24 March 2024) to read the cloned fragments using the universal M13-F/R primers. The chromatograms were examined using SnapGene Version 7, with the low-quality reads being trimmed before assembly and sequence extraction in the FASTA format. 

### 2.4. Phylogenetic Analysis of the D-Loop Region

To identify suitable D-loop sequences from various sheep breeds for comparison, we used Phan Rang sheep D-loop sequences to conduct a BLAST search on NCBI GenBank for the full D-loop or complete mtDNA sequences with clearly defined breed information as specified by the submitters. These sequences were then used as queries to perform an additional BLAST search to identify homologous sequences with the highest sequence identity. The breed labels of these highly identical homologous sequences were compared with those of the queries to ensure consistency. For the final analysis, only sequences with consistent breed labeling were selected. Additionally, we incorporated the D-loop sequences from Lv et al. (2015) [3] in the analysis. A comprehensive list of sheep breeds and their accession numbers is included in Supplementary Table S1.

The sequence alignment was performed using the MUSCLE algorithm within the MEGA XI suite [36,37]. The resulting alignments were further refined using Jalview Version 2 [38]. To facilitate the downstream processes, these alignments were saved in multiple file formats, including FASTA, MEGA, and NEXUS. The Bayesian phylogenetic tree was reconstructed with BEAST version 2.7 [39], employing the HKY substitution model and the Coalescent Bayesian Skyline tree prior. The Markov chain Monte Carlo (MCMC) search was set at 10,000,000 generations with a burn-in rate of 10%. The phylogenetic trees were visualized and refined using the iTOL web tool (https://itol.embl.de/, accessed on 24 March 2024) [40].

### 2.5. Statistical Analyses of the D-Loop Data

To determine the mtDNA haplogroup identity of Phan Rang sheep, we employed the methodology outlined by Tarlykov et al. (2021) [41]. We computed the haplogroup number (Nh), haplotype diversity (h), nucleotide diversity (π), and the average number of nucleotide differences (K) using DnaSP v6.12 [42]. The haplotype analysis by DnaSP was used to construct the Median Haplotype Network through POPART [43]. The demographic history of the population was assessed through pairwise mismatch distribution and neutrality tests (Tajima’s D, Fu’s Fs, and Fu and Li’s D and F tests) using both DnaSP and Arlequin 3.5.2 [44], and the mismatch distribution chart was plotted using Microsoft Excel.

The demographic dynamics of the Phan Rang sheep were further examined by constructing a Bayesian Skyline Plot (BSP) via BEAST 2.0, using a stepwise constant function. We opted for HKY + G + I nucleotide substitution model, running each MCMC simulation for 200 million generations and sampling every 1000 generations. The initial 10% of the samples were discarded as the burn-in period. In the absence of a specific mutation rate for the sheep D-loop, we utilized the cattle mtDNA D-loop mutation rate of 6.97 × 10^−7^ per site per year for calibration [45]. The final BSP was visualized using TRACER v1.7.2 [45], focusing on a time frame starting approximately 8 thousand years ago.

### 2.6. Y Chromosome Marker Haplotype Assignment

The chromatogram sequencing files for the SRY and SRYM18 markers were examined closely in both forward and reverse directions to identify specific substitutions (g88 in SRY), the number of repeats, and the total length of SRYM18 that together define the Y chromosome haplotypes. The designation of Y chromosome haplotypes in Phan Rang sheep followed established protocols, as detailed by Meadows et al. (2009) [28] and Wang et al. (2015) [30].

## 3. Results

### 3.1. Morphometric Features

**Figure 1:**
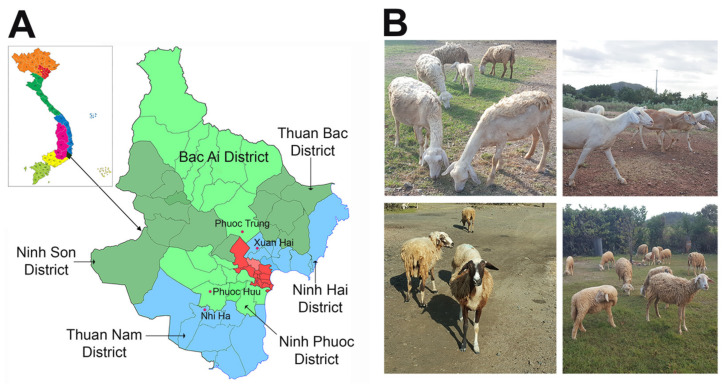
Sampling locations and representative individuals of Phan Rang sheep. (**A**) The map shows the location of Ninh Thuan province (inset) in Vietnam, the enlarged map of Ninh Thuan, and the communes where sheep herds were sampled for both morphometric data and blood collection. (**B**) Images of Phan Rang sheep taken from February 2022 to February 2023 show several distinct morphological features, suggesting multiple origins of Phan Rang sheep.

Despite some variability in physical traits, Phan Rang sheep typically exhibit long, slender faces with a light Roman nose and slightly drooping ears. While the predominant coat color is white, individuals with brown, black, or white coats with brown patches (piebald) are also present. Figure 1 provides detailed representations of the geographical locations where the Phan Rang sheep were sampled, as well as an illustration of some variations and characteristics of their physical traits.

Table 1 shows the morphometric data of the Phan Rang sheep, detailing observations from a cohort of 30 ewes and 30 rams; all are regarded as the breeding stock of the herds. Our findings on weight, body length, wither height, and heart girth generally concur with previous findings [12,13]. However, our results for tail length, averaging 35.2 ± 4.6 cm in rams and 27.3 ± 3.0 for ewes, diverge significantly from those reported by Doan et al. (2006) [13], who noted an average tail length for both sexes of only 28 cm. Additionally, the data on head length, head width, sternum height, and cannon circumference were reported for the first time.

The majority of the sheep being measured, 90%, exhibit white coats with fine fleece, while the remaining 10% also have white coats but with coarse fleece. The survey data from the households involved in this study showed that over 90% of Phan Rang sheep possess white coats, while brown and piebald are less common, at under 10%. The results somewhat deviate from Doan et al.’s (2006) [13] findings, which reported that 83.5% of Phan Rang sheep had white coats and 16.5% had brown or white–brown coats. Regarding wool texture, the ratios reported previously [13] were 86.5% and 13.5% for fine fleece and coarse fleece, respectively. 

### 3.2. Phylogenetic Relationships of Phan Rang Sheep Based on the D-Loop Data

The PCR amplification of the full D-loop region consistently produced fragments of approximately 1.3 kbp in length. The subsequent Sanger’s sequencing led to the generation of forward and reverse chromatograms, which were trimmed and merged to construct the final, polished sequences with lengths varying from 1092 to 1180 bp. The sequences were subjected to a BLAST search to screen for potential NUMTs contamination. Sequences exhibiting less than 98% identity with the D-loop sequences from contemporary world sheep breeds, as indicated by Nguyen et al. (2024) [46], were excluded to ensure authenticity. Colonies screening was repeated until sequence identity by BLAST search was greater than 98% to isolate the genuine D-loop sequences. 

**Figure 2:**
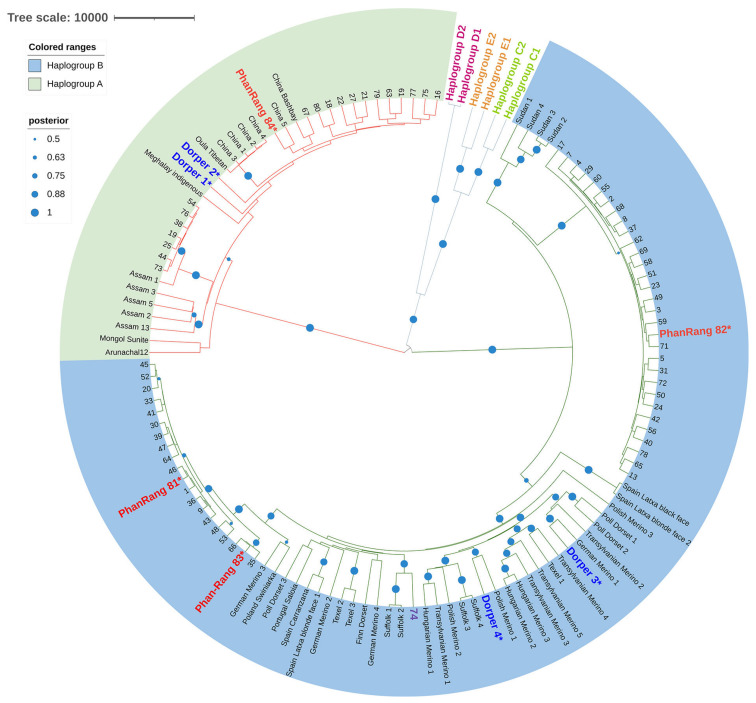
The Bayesian phylogenetic tree shows a relationship between Phan Rang sheep and some of the world’s sheep breeds. Posterior probability was used to test the robustness of the tree. The Phan Rang sheep from Ninh Thuan are labeled using numerical symbols based on the sampling order, while the Phan Rang sheep from the research center were noted with an additional asterisk. The accession numbers of the sheep breeds used in the phylogenetic analysis are listed in Supplementary Table S1. The stock breed sheep from the research center (Phan Rang and Dorper ewes) were included to verify the authenticity of the Phan Rang sheep from Ninh Thuan. Haplogroup B is represented by light blue, while haplogroup A is represented by light green. Other haplogroups were included to provide a complete picture of all the mitochondrial haplogroups. The tree scale bar represents the average number of substitutions per site across the whole tree. The posterior probability of each branch was represented as dots with sizes proportional to the probability values.

The near-complete or complete D-loop sequences from 19 rams and 49 ewes from four districts in Ninh Thuan were used for the phylogenetic analysis. Additionally, the sequences from four purebred adult Phan Rang and four purebred Dorper ewes from the Son Tay Goat and Rabbit Research Center were included to affirm the authenticity of the samples collected in the field. The data set was supplemented with the D-loop sequences from a study by Lv et al. (2015), particularly the sequences representing different mitochondrial haplogroups [3]. The resulting phylogenetic tree, depicting the relationship between Phan Rang sheep and various global sheep breeds, is presented in Figure 2.

The phylogenetic analysis based on the full D-loop region reveals two major clades for Phan Rang sheep. One clade belongs to mtDNA haplogroup A and clusters with sheep breeds from India, Mongolia, Tibet, and China; the other clade, belonging to haplogroup B, clusters with well-known European breeds like Suffolk and Merino. Notably, several haplogroup B Phan Rang sheep form their own cluster that does not closely associate with any breeds under investigation, suggesting unique genetic characteristics. 

**Figure 3:**
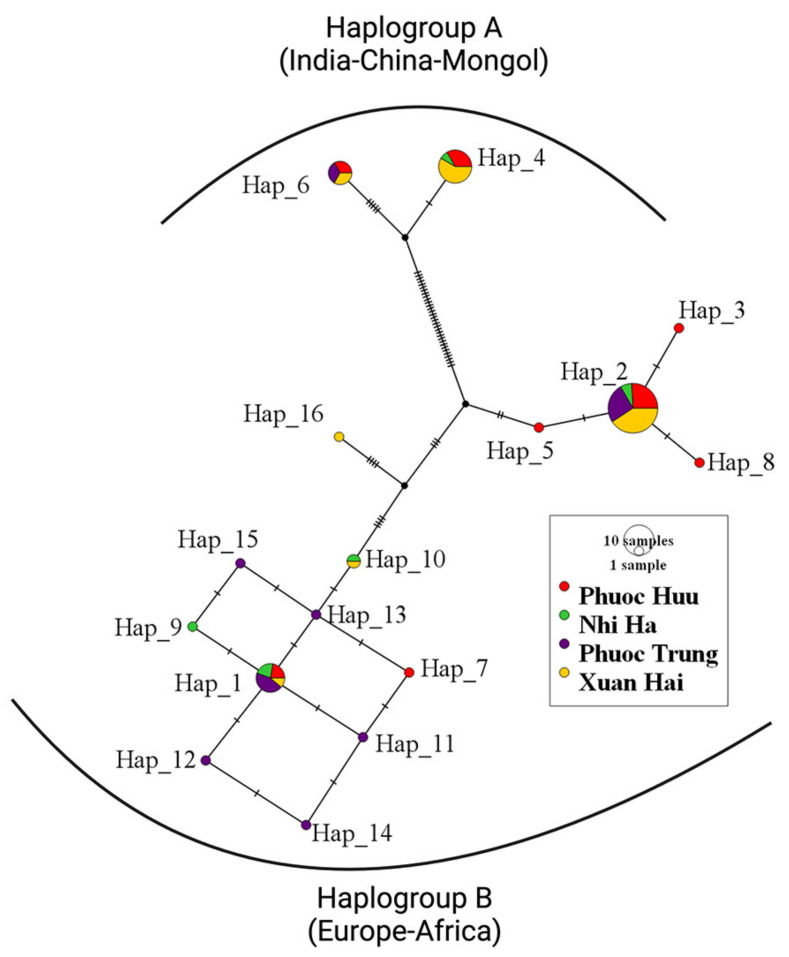
Median−joining haplotype network of the Phan Rang sheep from Ninh Thuan, showing the mtDNA haplotypes’ relationship. The sequences of the Phan Rang sheep raised at the research center were omitted.

To further examine the genetic diversity within these haplogroups, we constructed a median-joining haplogroup network based on the identified D-loop haplotypes (Figure 3). The network revealed sixteen distinct haplotypes within the Phan Rang sheep population in Ninh Thuan, with only two belonging to haplogroup A and the remaining fourteen belonging to haplogroup B.

### 3.3. Sequence Variability and Diversity Analysis of the Phan Rang Sheep

Table 2 presents the findings of the genetic diversity indices calculated for Phan Rang sheep D-loop sequences. The diversity analysis reveals a total of 16 haplotypes with a haplotype diversity of 0.79 and a nucleotide diversity of 0.0177. These values indicate a moderate to low diversity in both haplotype and nucleotide diversity. Only the D-loop sequences of Phan Rang sheep from Ninh Thuan were included in the analysis. 

### 3.4. Dynamic and Historical Profile of the Phan Rang Sheep

**Figure 4:**
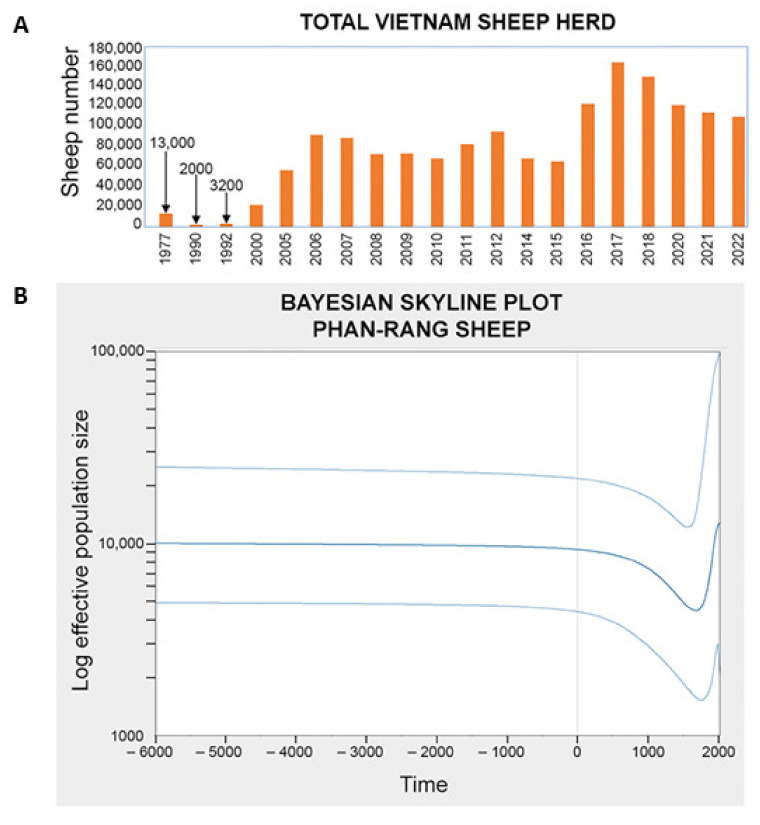
Population dynamics of the Phan Rang sheep by statistics and by simulation. (**A**) Statistics of the Phan Rang sheep population through time. The data from 1977 are from Mason IL. Prolific tropical sheep. Food and Agriculture organization of the United Nations. 1981 (Accessed on 12 March 2024) (http://www.fao.org/DOCREP/004/X6517E/X6517E00.HTM). The data from 1990 to 2000 are from Le M.C.; Le D.D. Sheep raising techniques (in Vietnamese). Vietnam Agriculture Publishing House: Hanoi, Vietnam, 2005; pp. 12–14. The data from 2000 to 2012 are from Bui V.L. Assessment of Phan Rang sheep’s adaptability to Thua Thien Hue raising conditions. Doctoral dissertation, Hue University, Hue—Vietnam. 2014. The data from 2013 to the present day are from Vietnam livestock statistics: Vietnam national livestock statistic reports (Accessed 2 April 2023). http://channuoivietnam.com/thong-ke-chan-nuoi/. The data for some years were missed or could not be retrieved. (**B**) The BSP modeling the population dynamic of Phan-Rang sheep for both haplogroups through time (from–6000 BC to present).

We employed neutrality tests such as Tajima’s D, Fu’s Fs, and Fu and Li’s D and F tests to assess the Phan Rang sheep population’s genetic diversity. All the tests yielded positive values for the haplogroups and the entire samples, as shown in Table 2. All the tests yielded positive results for haplogroup B but were not statistically significant. For haplogroup A, Fu’s Fs test suggested a bottleneck event with recent expansion, while F and Li’s D tests also suggested a bottleneck event but were insignificant. Combining two populations, these tests indicate a compelling bottleneck event followed by population expansion [47,48,49]. A survey of the population dynamics of the Phan Rang sheep from 1977 to the present showed evidence of a bottleneck during the 1990s. Figure 4A presents the dynamics of the Phan Rang sheep. To boost the credibility of the bottleneck event, we employed Bayesian Skyline Plots (BSPs) to model changes in the effective population size over time. The BSP plot (Figure 4B) depicts a significant decline in population size around the 18th to 19th centuries, followed by a recovery to present-day levels.

### 3.5. Lineages of the Phan Rang Male Sheep

**Figure 5:**
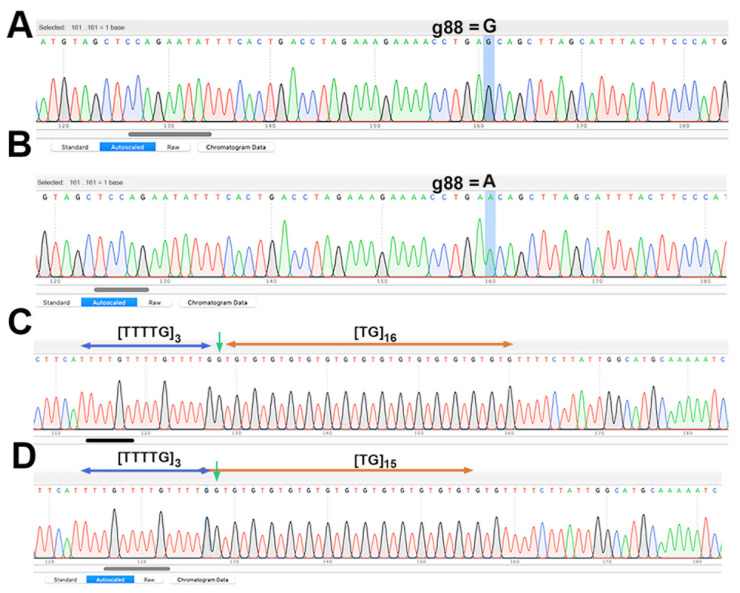
Chromatograms of the SRY and SRYM18 regions of the Phan Rang sheep. (**A**) Chromatogram of SRY with the G SNP genotype; (**B**) chromatogram of SRY with the A SNP genotype; and (**C**,**D**) chromatogram of the SRY18M repeat region.

To investigate the paternal lineages of the Phan Rang sheep, we analyzed two regions of the Y chromosome: the SRY and SRYM18 microsatellite markers. We sequenced these regions in 19 rams from our sample. The chromatogram analysis (Figure 5) revealed two distinct Y chromosome haplotypes, H5 and H6. The rams with the H6 haplotype displayed a specific combination of features: 15 repeats of the TG motif in the SRYM18 marker, a total length of 143 bp, and an Adenine (A) at position 88 of the SRY gene (g88). In contrast, the rams with the H5 haplotype only differed in having guanine (G) at position 88 of the SRY gene, 16 repeats of the TG motif, and a total length of 145 bp in the SRYM18 marker [28,30]. Three Phan Rang rams belonged to haplotype H5, while the remaining rams belonged to haplotype H6. 

## 4. Discussion

The Phan Rang sheep, an economically and culturally significant breed with potential value for climate change livestock adaptation, is facing a critical threat due to uncontrolled crossbreeding with imported sheep [15]. While this crossbreeding was initially intended to address inbreeding, the rapid expansion of hybrid offspring in Ninh Thuan and Binh Thuan has raised concerns about the dilution and potential loss of the purebred Phan Rang’s unique genetic heritage. To effectively conserve this sheep breed, comprehensive information on the breed is urgently needed. However, such data are currently unavailable.

**Figure 6:**
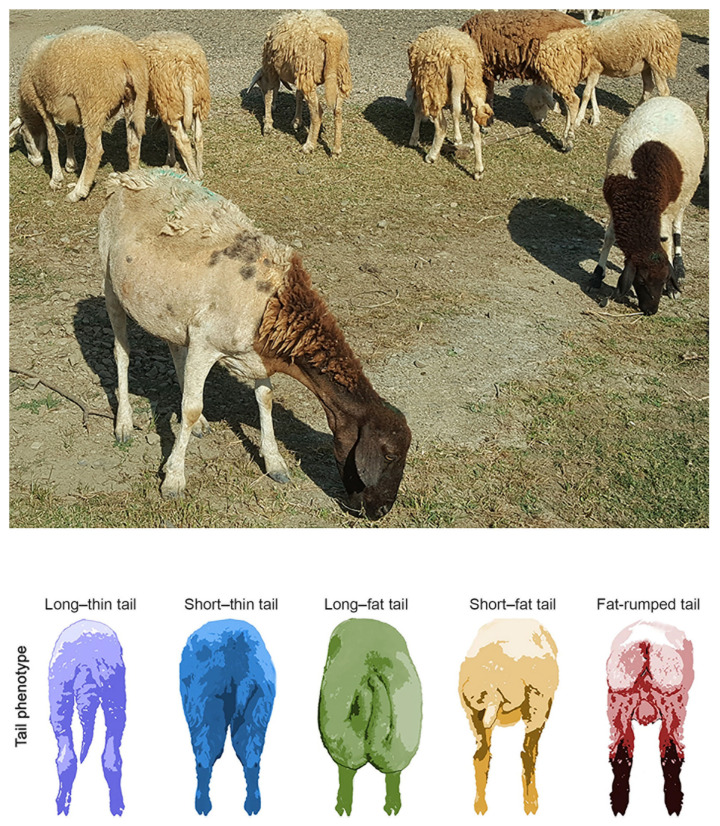
Phan Rang sheep tails. The lower panel is adopted from Kalds P. et al. (2022) [45]. The two individuals in the front with a black head and neck are likely hybrid sheep.

A fundamental challenge in conserving the Phan Rang sheep lies in the accurate identification of purebred Phan Rang sheep individuals. The existing data on morphological features [12,13] have proven insufficient due to their qualitative nature as well as their comprehensiveness. Our study aimed to supplement and update this information by analyzing the body conformation and morphological characteristics of the Phan Rang sheep. Our findings on body conformation largely align with previous studies, except for tail length (Figure 6). However, we concur with the conclusion from Doan et al., 2006 [13] that Phan Rang sheep belong to short, thin-tailed breeds [50]. The discrepancies in the coat colors and fleece types could be tentatively explained by two possible scenarios. First, the local preference for white-coated sheep, driven by a belief in their superior heat tolerance [13], could have skewed the sample composition. Alternatively, the limited sample size might not fully capture the breed’s coat color variation. Additionally, we collected measurements for previously unreported features like head length, head width, sternum height, and cannon circumference. While the potential limitations of our small sample size warrant further investigation, the data are expected to serve as a valuable source for quick identification of purebred Phan Rang sheep.

Our analysis of 68 D-loop sequences representing the Phan Rang sheep population in Ninh Thuan revealed a breakdown of haplotypes by sex. Among the nineteen rams sampled, eight belonged to haplogroup A, associated with Asian origins, while the remaining eleven belonged to haplogroup B, related to European origins. Similarly, the ewes displayed a distribution skewed towards haplogroup B, with 38 individuals compared to the 11 belonging to haplogroup A. These findings provide genetic evidence supporting the hypothesis of dual maternal origins for the Phan Rang sheep, aligning with the records on the breed [7]. The median-joining haplotype network revealed four prominent haplotypes (2, 4, 1, and 6), with the remaining 13 as singletons. We excluded the D-loop of the Phan Rang sheep raised at the research center from the haplotype network analysis to avoid anomalies that could not be sufficiently accounted for.

Based on the D-loop sequences, most Phan Rang sheep belong to haplogroup B, commonly found in European breeds, while the remaining sheep fall under haplogroup A, dominant in South Asia and East Asia. The individual number 74 deviates from the expected pattern, displaying a closer relationship with the European breeds than other Phan Rang sheep in the study, suggesting possible crossbreeding with the Dorper sheep.

The inclusion of the purebred Phan Rang sheep (marked with asterisks) alongside purebred Dorper sheep from the research center as a reference supports the authenticity of our Phan Rang sheep samples collected in Ninh Thuan, despite the limited number of sampling sites. The high prevalence of hybrid sheep is the most concerning factor during the sampling process. Our precautions of selecting households and sheep individuals using stringent criteria have ensured reliable mitochondrial data for the phylogenetic analysis. Extra precaution was also taken to avoid possible NUMTs contamination, which could have compromised the findings.

Our investigation into the paternal origins of Phan Rang sheep revealed two potential ancestral sources. Most rams carry the H6 haplotype, which is prevalent in Eurasia. The remaining rams belong to the H5 haplotype, commonly found in the sheep populations of Russia, Eastern Europe, and South West China [14]. Interestingly, all the rams carrying the H5 haplotype belonged to haplogroup B based on their D-loop analysis (discussed previously). On the other hand, the rams with the H6 haplotype were more evenly distributed across both haplogroups A and B. The evidence from the Y chromosome further strengthens the duo origin hypothesis.

While the world’s most common haplogroups are present in our Phan Rang sample, the overall genetic diversity appears significantly lower compared to findings from similar studies such as Kamalakkannan R. et al. (2021) [27] or Salim B. et al. (2023) [45]. The observed diversity aligns more closely with the findings reported by Mousumee M.A. et al., 2020 [23]. The observed difference might be explained by the nature of the sheep population under investigation. Sheep breeds like those from India and Sudan have a long history in their native regions, potentially leading to higher genetic diversity. Conversely, the recent import of sheep to Bangladesh, similar to the Phan Rang sheep, might account for a lower level of overall genetic diversity due to the low number of the founding populations.

**Figure 7:**
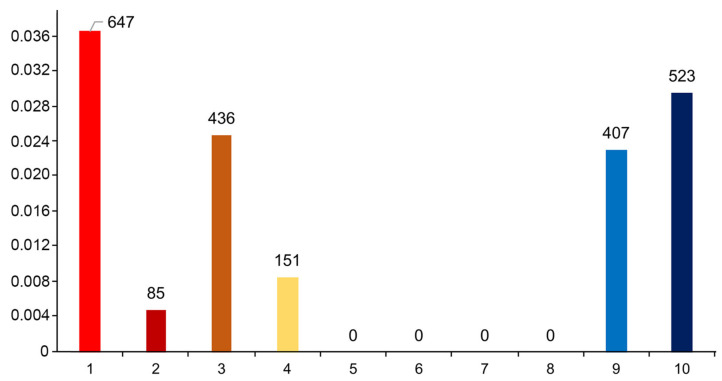
Mismatch distribution plot based on the D-loop data.

The inferred bottleneck event from the BSP plot seems to match up well with a recorded drop in the population of the Phan Rang sheep during the 1990s. However, the mismatch distribution plot (Figure 7) does not support the hypothesis of recent population expansion. This could mean other factors have contributed to the increase in the population, such as sheep imports, which could be hidden from the analysis due to the inheritance nature of mitochondrial DNA markers.

## 5. Conclusions

In conclusion, this study represents the first genetic analysis of the Phan Rang sheep, a breed of significant economic and cultural value to Vietnam. Our findings, based on the mitochondrial DNA (D-loop) and Y chromosome markers, provide strong support for the hypothesis of dual origins for the Phan Rang sheep, with contributions from both Asian and European lineages. Additionally, this study provides comprehensive morphometric information for the identification of the breed, which is expected to serve as a foundation for future work on the breed.

Despite the limitations, the results offer a crucial starting point for future research on Phan Rang sheep, which aims to address important questions such as population structure and the genetic basis for valuable traits such as heat resilience. 

## Figures and Tables

**Table 1:** Morphometric measurements of Phan Rang sheep.

Morphometrics	Male (30)		Female (30)	
	Mean ± Standard Deviation	Coefficient of Variance	Mean ± Standard Deviation	Coefficient of Variance
Body weight (kg)	41.8 ± 4.5	10.8	33.3 ± 4.6	13.9
Body diagonal length (cm)	72.6 ± 3.7	5.1	66.1 ± 3.3	5.0
Sternum height (cm)	41.9 ± 3.3	7.8	38.2 ± 1.8	4.7
Chest width (cm)	68.1 ± 3.3	4.9	62.2 ± 3.3	5.3
Heart girth (cm)	17.9 ± 1.7	9.7	12.8 ± 0.5	4.2
Tail length (cm)	35.2 ± 4.6	13.0	27.3 ± 3.0	13.9
Head length (cm)	22.9 ± 1.7	7.5	20.3 ± 1.3	6.6
Head width (cm)	11.4 ± 0.7	6.2	10.3 ± 0.5	4.6
Ear length (cm)	16.3 ± 2.5	15.3	13.1 ± 1.1	8.7
Cannon circumference (cm)	11.9 ± 1.3	10.8	10.6 ± 0.7	6.7

**Table 2:** Genetic diversity analysis of the Phan Rang sheep from Ninh Thuan based on their D-loop.

	MtDNA Haplogroup B	MtDNA Haplogroup A	Total
Sample size	49	19	68
Number of polymorphic sites	19	6	50
Number of haplotypes	14	2	16
Haplotype diversity	0.67 ± 0.0046	0.49 ± 0.0046	0.79 ± 0.0041
Mean of nucleotide differences	6.14	2.95	18.86
Nucleotide diversity	0.00576 ± 0.0000002	0.00276 ± 0.0000001	0.01770 ± 0.0000020
Fu’s Fs statistic	0.209 (0.140)	7.096 (0.009)	9.676 (*p* > 0.01)
Fu and Li’s D test statistics	0.13395 (*p* > 0.1, not significant)	1.25359 (0.1 > *p* > 0.05, not significant)	1.69095 (*p* < 0.02)
Fu and Li’s F test statistics	0.67502 (*p* > 0.1, not significant)	1.78939 (*p* > 0.01)	2.42545 (*p* > 0.01)
Tajima’s D test	1.40120 (*p* > 0.1, not significant)	2.32066 (*p* < 0.05)	2.56444 (*p* < 0.05)

## Data Availability

Data are available upon request.

## Supplementary Materials

The following supporting information can be downloaded at https://www.mdpi.com/article/10.3390/ani14142020/s1. Table S1: A list of the sheep breeds and their GenBank accession numbers used in the phylogenetic analysis; and Table S2: The primer sequences used in this study.
