# Supplementary material for: Characterizing the Phan Rang Sheep: A First Look at the Y Chromosome, Mitochondrial DNA, and Morphometrics
Source: Animals (Basel). 2024 Jul 9;14(14):2020. doi: 10.3390/ani14142020 (PMC11274324; doi:10.3390/ani14142020)
Supplement: Supplementary file 1 [file animals-14-02020-s001.zip › animals-3019057-supplementary.pdf]

Supplementary Table S1. List of sheep breeds and their GenBank accession numbers used in the phylogenetic analysis.

No	Sheep breed name	Accession number	Reference
1	Meghalay indigenous	ON148439.1	This study
2	Assam 1	ON110809.1	This study
3	Assam 2	ON110806.1	This study
4	Arunachal12	ON148444.1	This study
5	Assam 3	ON110804.1	This study
6	Assam 4	ON110811.1	This study
7	Assam 5	ON110810.1	This study
8	Assam 13	ON110805.1	This study
9	Oula Tibetan	KU575248.1	This study
10	China 1	KX344226.1	This study
11	China 2	KX344219.1	This study
12	China 3	KX344215.1	This study
13	China 4	KX344214.1	This study
14	China 5	KX344158.1	This study
15	Sudan 1	LC456426.1	This study
16	Sudan 2	LC456430.1	This study
17	Sudan 3	LC456503.1	This study
19	Sudan 4	LC456478.1	This study
19	Polish Merino 1	JN574051.1	This study
20	Polish Merino 2	JN574052.1	This study
21	Polish Merino 3	JN574053.1	This study
22	Spain Latxa blonde face 1	KT879152.1	This study

23	Spain Carranzana	KT879075.1	This study
24	Spain Latxa black face	KT879055.1	This study
25	Spain Latxa blonde face 2	KT879034.1	This study
26	Portugal Saloia	DQ491693.1	This study
27	Transylvanian Merino 1	JN574075.1	This study
28	Transylvanian Merino 2	JN574076.1	This study
29	Transylvanian Merino 3	JN574078.1	This study
30	Transylvanian Merino 4	JN574077.1	This study
31	Transylvanian Merino 5	JN574080.1	This study
31	German Merino 1	JN573942.1	This study
32	German Merino 2	JN573943.1	This study
33	German Merino 3	JN573944.1	This study
34	German Merino 4	JN573945.1	This study
35	Hungarian Merino 1	JN574010.1	This study
36	Hungarian Merino 2	JN574011.1	This study
37	Hungarian Merino 3	JN574012.1	This study
38	Texel 1	AY829406.1	This study
39	Texel 2	AY829407.1	This study
40	Texel 3	AY829410.1	This study
41	Poll Dorset 1	DQ320067.1	This study.
42	Poll Dorset 2	DQ320065.1	This study
43	Poll Dorset 3	AY829381.1	This study
44	Suffolk 1	MK174670.1	This study
45	Suffolk 2	MK174671.1	This study

46	Suffolk 3	MK174672.1	This study
47	Suffolk 4	MK174674.1	This study
48	China Bashbay	KF938330.1	Lv H.F. et al. 2015
49	Poland Swiniarka	KF938349.1	Lv H.F. et al. 2015
50	Finn Dorset	EF490451.1	This study
51	Mongolian Sunite	KF938317.1	Lv H.F. et al. 2015
52	Haplogroup D1	HM236180.1	Lv H.F. et al. 2015
53	Haplogroup D2	HM236181.1	Lv H.F. et al. 2015
54	Haplogroup E1	HM236182.1	Lv H.F. et al. 2015
55	Haplogroup E2	HM236183.1	Lv H.F. et al. 2015
56	Haplogroup C1	HM236178.1	Lv H.F. et al. 2015
57	Haplogroup C2	HM236179.1	Lv H.F. et al. 2015
58	Meghalay indigenous	ON148439.1	This study

The accession numbers for Phan-Rang sheep D-loop can be found on NCBI with the accession number: OR683520 to OR683588.

Supplementary Table S2. Primer sequences used in this study. Primer positions on mtDNA are indicated in the bracket.

Name	Sequences (5′ → 3′)
D-loopF (15338-15358)	CCAGAGAAGGAGAACAACCAA
D-loopR (21-40)	GCATTTTCAGTGCCTTGCTT
SRY-F	TCAGTAGCTTAGGTACATTCA
SRY-R	GTGCTACATAAATATGATCTGC
SRYM18-F	GGCATCACAAACAGGATCAGCAAT

SRYM18-R	GTGATGGCAGTTCTCACAATCTCCT
